# Association of adherence to the dietary approach to stop hypertension diet and diet quality indices among women in Tehran: A cross- sectional study

**DOI:** 10.15171/hpp.2019.40

**Published:** 2019-10-24

**Authors:** Hana Arghavani, Elnaz Daneshzad, Nazli Namazi, Bagher Larijani, Mohammadreza Askari, Nick Bellissimo, Katherine Suitor, Leila Azadbakht

**Affiliations:** ^1^Department of Community Nutrition, Tehran University of Medical Sciences, Tehran, Iran; ^2^Obesity and Eating Habits Research Center, Endocrinology and Metabolism Molecular-Cellular Sciences Institute, Tehran University of Medical Sciences, Tehran, Iran; ^3^Endocrinology and Metabolism Research Center, Endocrinology and Metabolism Clinical Sciences Institute, Tehran University of Medical Sciences, Tehran, Iran; ^4^School of Nutrition, Ryerson University, Toronto, Canada; ^5^Diabetes Research Center, Endocrinology and Metabolism Clinical Sciences Institute, Tehran University of Medical Sciences, Tehran, Iran; ^6^Department of Community Nutrition, Isfahan University of Medical Sciences, Isfahan, Iran

**Keywords:** Dietary approaches to stop hypertension, Diet records, Diet therapy

## Abstract

**Background:** Examining dietary approach to stop hypertension (DASH) diet based on other dietary quality indices can be helpful to clarify positive aspects of this healthy dietary pattern. We aimed to examine the association between the DASH diet score and some diet quality indices among Iranian women.

**Methods:** In this cross-sectional study, 304 women aged 20 to 50 years old were recruited. Dietary diversity score (DDS), dietary energy density (DED), adherence to DASH diet, AlternativeHealthy Eating Index (AHEI) and mean adequacy ratio (MAR) were examined as suggested by previous articles. Dietary quality indices, anthropometric indices, and dietary intake were categorized based on DASH tertiles. A semi-quantitative food frequency questionnaire with 168items was used for dietary assessment.

**Results:** There were no significant differences in the demographic characteristics of participants across DASH tertiles (P>0.05). Participants who adhered more to the DASH diet had lower DEDthan those with lower adherence (0.99±0.35 vs 1.26±0.30; P=0.01). Significant differences were observed in the index of DDS across tertiles (P=0.01), however no differences in nutrient adequacy ratio (NAR) and MAR (0.93) index across the DASH categories were found.Additionally, DDS to DED in the top tertile of the DASH diet was greater than the bottom one(6.7±2.9 vs 4.4±1.9; P=0.001).

**Conclusion:** The present study indicated that greater adherence to the DASH diet is inversely associated with DED and AHEI. As well as, there was a positive association between the DASHdiet and DDS/DED ratio. However, more studies are needed to confirm the results of this study

## Introduction

DASH (Dietary approach to stop hypertension) diet is one of the healthy dietary patterns that was established for patients with hypertension in the 1990s.^1^ The DASH diet is characterized by a high amount of fiber including fruits, vegetables, and whole grains and a low intake of sodium and sweets while emphasizing the consumption of low-fat dairy products, white meat, and nuts.^1^ Adherence to the DASH dietary pattern has demonstrated beneficial effects on the treatment of hypertension, cardiovascular diseases, type 2 diabetes mellitus and metabolic syndrome.^2-4^ Indeed, previous studies have shown that nutrition status is closely related to the inception of these diseases.^5-8^ To clarify other positive aspects of this healthy diet, examining the DASH diet based on other dietary quality indices will be helpful.

Several indices of healthy eating have been developed to reveal the level of adherence to a specified dietary pattern in the last three decades. They provide an approach which assesses diet quality as a whole compared to the less preferred method of individual nutrient content and the consideration of single foods.^9^ Diet quality refers to both food variety and nutrient adequacy.^10^ Previous studies indicated that the dietary diversity score (DDS), Alternative Healthy Eating Index (AHEI), nutrient adequacy ratio (NAR) and mean adequacy ratio (MAR), as well as dietary energy density (DED), are suitable indicators for diet quality assessments.^11,12^ DDS focuses on the variety of food intake to reduce mortality and morbidity.^13^ Furthermore, it has been shown that DDS is a good indicator for the adequacy of some micro-nutrients. Prior studies reported that DDS is associated with cardiovascular risk factors,^14^ obesity, abdominal adiposity,^15^ and metabolic syndrome.^12,16^ Other diet quality indices emphasize the consumption of healthy foods and the restriction of less healthy ones.^12,17^

Due to the previous research in Tehran it is evident that overall diet quality status of Tehranian women needs to be improved. Based on the 2015 Dietary Guidelines, the DASH dietary pattern is an example of a healthy eating pattern that can be considered practical and accessible to the public. While the relationship between the DASH diet and chronic metabolic disease has been studied,^18-20^ the association between the DASH diet and other dietary quality indices is not well understood. Specifically, the DASH diet has not been investigated regarding the overall diet quality status of women in Iran. The present study aimed to assess the association between the DASH diet score and diet quality indices among Iranian women.

## Materials and Methods

### Study design and participants

In this cross-sectional study, 304 women referred to 10 health care centers in Tehran, Iran were recruited from October 2016 to March 2017. A representative sample of women aged 20 to 50 years was selected by a multistage cluster random sampling method. The number of women to be sampled from each health care center was calculated according to the proportion of participants referred to each center. All health centers were located in the south of Tehran; no differences in socioeconomic status between these centers was observed. Women residing in Tehran with Iranian nationality who did not follow a specific diet were included. Exclusion criteria were as follows: a history of diabetes, cardiovascular disease, liver or kidney disorders, cancer, pregnant and/or lactating women, and women who had total diet energy intake (kcal/day) outside the range of 800 to 4200 kcal/day. The Alternated Healthy Eating Index score was considered the main dependent variable for calculating the sample size (16). A sample size of 248 was determined using the formula N = [(Z_1-α/2_)^2^×(S)^2^]/d^2^ considering “α” = 0.05, “d” = 4%, and the effect size = 1.5. In order to compensate for the potential exclusion of participants due to under- and over-reporting of total energy intake, 304 participants were selected for inclusion. All eligible participants signed written informed consents at the baseline.

### Anthropometric assessment

Using a digital scale (Seca, Hamburg, Germany), weight was measured in minimally clothed participants without shoes. Height was measured using standard protocol with a tape measure. The body mass index (BMI) was calculated as weight (kilograms) divided by squared height (m^2^). Waist circumference (WC) was measured at the narrowest circumference using an outstretched tape measure. Hip circumference was measured at the maximum circumference over light clothing with a precision of 0.1 cm. All measurements were taken by the same technician to limit observer error.

### Physical activity

The International Physical Activity Questionnaire-short form (IPAQ) was used to assess physical activity level,^21^ where metabolic equivalent-hours per day (MET-h per day) was considered the unit for expressing this measure.

### Socioeconomic status

Socioeconomic status data was collected by a validated Persian socio-economic questionnaire which included measures of income, area of residency, number of family members, educational level, vehicle ownership, number of vehicles and their types (if applicable), real estate ownership or tenantship, and number of bedrooms.^22^

### Dietary assessment

A previously validated and reliable semi-quantitative Food Frequency Questionnaire (FFQ) with 168-items was used for evaluating dietary food intake.^23^ The FFQ contained a comprehensive list of commonly consumed foods and mixed dishes with standard serving sizes. The frequency and quantity of consumed food items were converted to grams per day and recorded on a daily, weekly, or monthly basis within the past year.^23^ For dietary analysis, Nutritionist IV software (First Databank Inc., Hearst Corp., San Bruno, CA, USA) modified for Iranian foods was used. All items were converted to daily intake values to calculate total energy intake.

### Calculation of dietary quality indices

*DASH score:* We calculated the DASH score to check participants adherence to the DASH diet based on foods and nutrients that were emphasized or summarized in the eight pillars of the DASH diet: low intake of sodium, whole grains, sweets, sugar-sweetened beverages and processed and red meats, and a high intake of low-fat dairy products, nuts and legumes, fruits and vegetables. A score of 4 was given to participants with the highest quartile of low-fat dairy products, vegetables, fruits, nuts, whole grains and legumes and a score of 1 was given to participants with the lowest quartile of aforesaid foods. Similarly, a score of 4 was given to participants within the lowest quartile of red and processed meats, sugar-sweetened beverages, sweets, and sodium consumption and a score of 1 for participants with a high consumption of these food categories. The DASH score was calculated for each participant by the summation of scores from the eight components for a total DASH score within a range of 8 and 32.^2^

*DDS:* The previously described method by Kant et al was used to calculate a DDS score.^24^ Briefly, five food groups (vegetables, bread and grains, meat, dairy, and fruit) were divided into twenty-three subgroups. A food item was considered a consumed when the participant had eaten at least one-half of a serving per day based on the food pyramid quantity. The score for each food group was calculated by the sum of subgroup category scores, and multiplied by 2. The total DDS ranged between 0 and 10.

*DED score:* The DED score was calculated by dividing each participant’s total energy intake by total grams of food per day. In consideration of the current ongoing debate whether to include or exclude beverages in the calculation of the DED score, we opted to exclude beverages and calculate the DED score as the sum of the weight of 154 foods (excluding beverages). Therefore, the effect of the DED score on body weight was attributed to food intake, and not beverages.^23^

*AHEI 2010 score:* To calculate AHEI score, 9 different components including vegetables, fruit, nuts and soy intake, the ratio of polyunsaturated to saturated fatty acids, the ratio of white to red meat, trans fats, alcohol, cereal fiber, and multivitamins. After calculating the energy-adjusted amounts of the aforementioned food items using the residual method, participants were scored based on quintile. The score of 0 and 5 reflected the first and fifths quintiles, respectively.^25^ Scores from the 9 components were summated, and an AHEI score between 7 and 45 was obtained. A higher score represented a stricter adherence to the AHEI dietary eating pattern.

*NAR and MAR scores:* Based on an individual’s intake of a nutrient as a percentage of the recommended intake expressed by the NAR, MAR was estimated which outlines the overall nutritional adequacy of a population. NAR was calculated by dividing the estimated daily individual intake of zinc, iron, calcium, vitamin C, vitamin D, vitamin B12, vitamin B2, vitamin B6, and vitamin A to their dietary reference intakes (DRIs) that are reported based on sex and age.^12^ Each NAR score is summed and divided by the number of nutrients and multiplied by 100 to indicate the percentage of nutrients where the recommended intake is met.

### Statistical analysis

The distribution of variables across the tertiles of DASH score was assessed using a Kolmogorov–Smirnov test and confirmed by histogram. Variables with normal and non-normal distributions were presented as mean ± SD and median (25^th^ percentile, 75^th^ percentile), respectively. General characteristics of participants, dietary intake and dietary quality indices were categorized based on tertiles of DASH diet score. Participants were categorized based on tertile cut-off points for the DASH diet as follows: T1: <18, T2: 18-21, T3: 21). To compare participants in different tertiles of the DASH score, analysis of variance (one-way ANOVA) and chi-square tests were used for quantitative and qualitative variables, respectively. For quantitative variables with non-normal distribution, the Kruskal-Wallis test was used. We adjusted for a variety of factors that could potentially confound the relationship between the calculated DASH score and diet quality indices in multivariate analyses. Energy intake, age, educational level, and income were considered as control variables in multivariate analyses. Total energy intake was adjusted to ensure that observed associations were independent of participant’s energy intake. Most food and nutrient intakes were associated with total energy intake and therefore may affect the quality of food.^26^ Measurement error was reduced in data collection phase through the use of the self-reported dietary assessment. Age, educational level, and income were previously determined.^27^ There was no significant correlation between these covariates and diet quality, however it is possible that residual effects remain on observed outcomes. All variables related to dietary intake (macro- and micronutrients, food groups) and dietary quality indices (DDS, AHEI, DED, NAR, MAR) were adjusted for total energy intake using an analysis of covariance (ANCOVA). A partial correlation was run to determine the interaction between DASH score and dietary quality while controlling for energy intake, age, educational level and income. All statistical analyses were performed using SPSS version 16 software (SPSS Inc., Chicago IL, USA). P-values less than 0.05 were considered statistically significant.

## Results

In this cross-sectional study, the study sample consisted of 304 women with a mean age of 32.04 (SD 8.63) years. All participants initially recruited for the study were characterized to have a normal total energy intake and were included in study analyses. No participants were excluded for under or over reporting energy intake. General characteristics and anthropometric indices of the participants across the tertiles of DASH score are indicated in Table 1. The lowest tertile presents the lowest compliance and the highest one corresponds to the highest compliance for DASH dietary pattern. DASH score tertiles (from the lowest to the highest) consisted of 108, 101 and 95 participants, respectively. As shown in Table 1, there were no significant differences in demographic characteristics (age, educational level, disease background, family history of diseases, and physical activity) and anthropometric indices (BMI and WC) among DASH categories. Although the prevalence of overweight/obesity (51.0% vs. 43.0%; *P* = 0.74) and abdominal obesity (48.0 vs. 40.0%; *P* = 0.65) were greater in the lowest tertile compared to the highest one, no significant differences were found among categories.

Dietary intake patterns of participants are outlined in Table 2. There was no significant difference in total energy intake (*P* = 0.13), carbohydrate (*P* = 0.73), protein (*P* = 0.86) and dietary fiber (*P* = 0.06) across the DASH tertiles. However, participants in the lowest tertile consumed a diet higher in fat compared to those in the highest tertile (71.10 (SD 24.53) vs. 57.64 (SD 23.76) g/day, respectively; *P* = 0.0001). Specifically, significant differences in fat consumption were indicated between first and third tertiles (*P*≤ 0.01) and the second and third tertiles (*P* = 0.004). Among main food groups, there were statistically significant differences observed in fruit, and dairy intake across DASH categories (*P* = 0.0001 for all variables). Significant differences were observed between first and second tertiles (*P* = 0.01) and first and third tertiles (*P* ≤ 0.01) for fruit consumption. Similarly, the significant differences indicated in dairy intake were observed between first and second tertiles (*P* = 0.04) and first and third tertiles (*P* ≤ 0.001). No significant differences were found between tertiles for meat (*P* = 0.19) or grain consumption (*P* = 0.76). As presented in Table 2, there were no significant differences in mineral (zinc, iron, and calcium) and vitamin (C, D, B12, B2, B6, and A) intake in participants with a higher adherence to the DASH dietary eating pattern compared to participants with a lower adherence (*P*> 0.05 in all variables).

Diet quality indices across the tertiles of DASH eating pattern are represented in Table 3 and Table 4. Although not significant, participants in the second tertile had the most diverse diet (mean score = 5.52 [SD 1.25]) compared to the first (mean score = 5.10 [SD 1.15]) and third (mean score = 5.47 [SD 1.01]) tertiles. However, a significant difference was only observed between the first and second (*P* = 0.01) and first and third tertiles (*P* = 0.01). Our findings indicate that the second tertile of the dairy DDS was significantly higher than both the first (*P* = 0.006) and third tertile (*P* = 0.003). Yet the DDS across fruit (*P* = 0.01) and vegetable (*P* = 0.001) groups significantly increased from the first to the third tertile. Significant differences in fruit intake were observed between first and third tertiles (*P* = 0.005) whereas significant differences for vegetables were observed between first and second tertiles (*P* = 0.03) and first and third tertiles (*P* = 0.001). There were no significant relationships indicated in the NAR of mineral or vitamins measured and this translated to no significant difference calculated in the MAR index across the DASH tertiles. A partial correlation assessment to determine the interaction between DASH score and dietary quality while controlling for energy intake, age, educational level and income represented in Table 5. A significant correlation between DASH score and Mediterranean score was observed (*P* = 0.001).

## Discussion

The DASH diet is a healthy dietary pattern that has previously demonstrated potential benefits in the treatment of chronic diseases such as Crohn’s disease and diabetes.^28^ Typically its’ beneficial effects are attributed to the intake of a combination of healthy food groups. To calculate DASH score, participants who consumed more fruits, vegetables, whole grains, and nuts received a greater score compared to participants with a higher intake of meat, sodium and sweetened beverages.^29^ Further examination of the healthy dietary pattern exhibited in the DASH diet compared to other dietary quality indices will provide constructive information that can translate into the value and utility of this dietary pattern as a treatment and prevention tool. To our knowledge, the current study is the first in which the association between DASH score and other dietary quality indices has been examined in a healthy population.

We found that there were no differences in anthropometric indices among women in the tertiles of the DASH diet. This is in agreement with the 2003-2012 National Health and Nutrition Examination Surveys that demonstrated that there was no significant correlation between DASH score, body weight and waist circumference in American children and adolescents.^30^ In contrast, Saneei et al reported that there was an inverse correlation between the higher range of DASH score and waist circumference.^4^ In the present study, no considerable differences were reported for total energy intake among the participants in the tertiles of the DASH diet; the level of physical activity were similar among tertiles. Due to similar total energy intake and physical activity, such findings were expected.

Participants with higher adherence to the DASH diet consumed less fat, while they reported more consumption of fruits, vegetables, and dairy products. However this did not translate into significant differences observed in independent nutrients across DASH score tertiles. There are no prior studies that compare the intake of specific nutrients across DASH score tertiles. However, Wang et al established that the DASH diet does in fact provide sufficient amounts of nutrients such as vitamin C, riboflavin, thiamin, vitamin B6, folate, niacin, vitamin B12, magnesium, calcium, zinc, copper, and iron^31^ while Azadbakht et al reported that mean intake of vitamin C, magnesium, potassium and calcium were higher in the DASH diet compared to a control diet included a macronutrient composition of 50%–60% carbohydrates, 15%–20% protein, 30% total fat and 5% simple sugars which was close to a standard Iranian dietary pattern.^18^

The present cross-sectional study indicated that a greater adherence to the DASH diet is inversely associated with DED and AHEI. However, there was no correlation between DASH diet adherence, MAR and NAR. Furthermore, participants in the top DASH tertile had higher DDS to DED ratio, while women in the second tertile had a more diverse diet. It is notable that we obtained weak correlations between DASH and other diet quality indices, whereas some of them were statistically significant. Studies with larger sample size are needed to clarify these associations.

As the DASH diet emphasizes the high intake of fruits and vegetables and low consumption of fat and sugar sources, such findings were expected. Some studies reported that DED was associated with an increased risk of developing diabetes. They also suggested that DED is an independent predictor for increased fasting insulin levels, and obesity.^32-34^ Participants in the second DASH tertile demonstrated the highest DDS. The DASH eating pattern is characterized by high amounts of fiber and protein, whole grains, fruits and vegetables, low-fat dairy products, fish, poultry, nuts and sufficient amounts of minerals.^1^ Indeed, participants with greater adherence to the DASH diet had the highest score of dietary diversity for fruits and vegetables. Our study was in line with earlier reports which indicate that participants with a higher DDS consumed more fruits and vegetables.^14,23,35^ Participants in the highest DASH tertile were characterized by a higher DDS to DED ratio. This relationship was further supported after adjusting for total energy intake. This index examines the effects of dietary diversity and energy density simultaneously, and therefore is more suitable than considering each index independently.

There was a negative partial correlation between AHEI and DASH score while controlling for energy intake, age and educational level r = -0.20, *P*≤ 0.001 which was statistically significant. Although both diets are considered as healthy dietary patterns, this significant difference may be attributed to the inclusion and scoring of different food items. Indeed, AHEI considers the ratio of white to red meat, dietary fiber, types of fat (PUFA/SFA and trans fatty acid), multi-vitamin intake and alcohol while the DASH score does not. Similarly, the DASH dietary pattern limits the amount of fat, compared to AHEI which emphasizes the classification of fat, but not the quantity.

Although the present study was the first to examine the association between the DASH diet and other dietary quality for the first time, there were several study limitations that should be identified. We could not clarify the cause and effect correlation due to the cross-sectional design. Therefore, prospective studies are needed to evaluate these associations with in a longitudinal study design. Although a major strength of the study includes all healthy female participants, because the participants were only women we are unable to extrapolate our findings to men. All Iranian women were recruited from the same city, and therefore the population may have been too homogeneous to find strong differences. As such, our study findings may not adequately reflect Iranian women in general. Using FFQ as a retrospective dietary assessment tool may cause misclassification, however efforts were made to employ an adapted and validated FFQ.

Our findings indicate that a greater adherence to the DASH dietary pattern is inversely associated with DED and AHEI scores. There was no significant interaction reported between following the DASH diet, MAR, and NAR. Furthermore, the DDS to DED ratio in the highest tertile was greater than the lowest one. Future studies are needed to evaluate the association between the DASH diet and diet quality indices over a longer period of time, and include a larger geographical recruitment region. Observations should be made in both genders in order to delineate any differences that may be attributed to sex. It is recommended for prospective studies to employ dietary assessment tool to reduce participant reporting errors. According to the results, participants who were more adherent to the DASH diet had higher intakes vegetable, fruit, and dairy food groups. There was a positive association between the DASH diet and DDS as an indicator of diet quality assessment. Based on our results the DASH dietary pattern can be considered as a high-quality healthy eating pattern. The promotion and introduction of the healthy eating patterns outlined in the DASH diet can ultimately have important beneficial applications to not only policy decisions but the treatment and prevention of chronic metabolic dysfunction.

## Ethical approval

The present study was approved by the ethics committee of Tehran University of Medical Sciences under approval number 94-04-161-31112.

## Competing interests

The authors declare that they have no competing interests.

## Funding

This study is supported by Tehran University of Medical Sciences (grant number: 94-04-161-31112) and the National Elites Foundation and Iran National Science Foundation (grant number: BN092).

## Authors’ contributions

LA and BL designed the study; NB, ED, HA and MA contributed to data collection; data analysis was done by NB and LA; results were interpreted by ED, LA and BL; the report was drafted by NB, BL, LA, HA, KS and MA; critical revision of the report for important intellectual content was done by all the investigators.

## Acknowledgments

We would like to express our thanks to the Tehran University of Medical Sciences for approving the project number 94-04-161-31112 and the National Elites Foundation and Iran National Science Foundation for financial support.

**Table 1:** General characteristics and dietary intake of the participants across the DASH tertiles

**Variables**	**DASH Diet**			***P*** **Value fortrend** ^a^
	**T1 (n = 108)**	**T2 (n= 101)**	**T3 (n =95)**	
Age (y)	31.88 ± 8.47^bb^	32.11±7.83	33.22±8.63	0.451
BMI (kg/mb)	24.27±4.94	24.97±4.94	24.78±4.19	0.553
WC (cm)	85.32±11.90	85.68±11.57	85.35±11.39	0.972
Overweight and obesity (%)	51	35	40	0.744
Abdominal obesity (%)	48	31	40	0.650
Physical activity (MET-h/d)	30.62±3.3	30.86±3.69	31.21±3.51	0.461
Educational level (%)				
Illeterate	5.3	8.3	5.6	0.775
Diploma	28.3	25	27.1	
Universal	66.4	66.7	67.3	
Disease background (%)				
Hypertention	0.88	2.38	0	0.242
Dislipidemia	1.76	3.5	6.56	0.191
Family disease history (%)				
Diabetes	17.69	17.85	13.08	0.551
Stroke	15.92	14.28	14.95	0.952
Multi-vitamin consumption	26.54	22.61	24.29	0.835

Abbreviations: DASH, dietary approach to stop hypertension; BMI, body mass index; WC: waist circumference. ^a^ Mean± SD. ^b^ One-way ANOVA for quantitative and chi-square for qualitative variables and <0.05 is significant.

**Table 2:** Comparision of dietary intake and food groups across the tertiles of the DASH diet

**Variables**	**DASH diet**			***P*** **value fortrend** ^a^	***P*** **value for comparison T1&T2** ^b^	***P*** **value for comparison T1&T3** ^b^	***P*** **value for comparisonT2&T3** ^b^
	**T1 (n = 108)**	**T2 (n= 101)**	**T3 (n =95)**				
Total energy intake (kcal/day)	2250 ± 658^c^	2209 ± 659	2069 ± 690	0.130	0.772	0.812	0.365
Carbohydrate (g/day)	335.15 ± 102.97	333.80 ± 98.24	324.20 ± 126.71	0.732	0.512	0.721	0.554
Fat (g/day)	71.10 ± 24.53	68.07 ± 26.12	57.64 ± 23.76	<0.001	0.333	<0.001	0.0041
Protein (g/day)	78.44 ± 26.09	77.06 ± 27.15	76.32 ± 35.27	0.865	0.814	0.513	0.615
Fiber (g/day)	71.10 ± 24.53	68.07 ± 26.12	57.64 ± 23.76	0.062	0.341	0.431	0.373
Food and food groups (g/day)							
Fruits	271.55 ± 162.48	341.48 ± 196.36	438.22 ± 244.90	<0.001	0.010	<0.01	0.035
Vegetables	267.31 ± 167.46	268.18 ± 129.54	312.14 ± 162.86	<0.001	0.553	0.435	0.712
Meats and alternatives	125.62 ± 62.40	114.63 ± 75.81	122.70 ± 68.69	0.193	0.271	0.195	0.451
Grains	475.85 ± 159.47	472.57 ± 153.74	462.12 ± 156.26	0.764	0.614	0.551	0.413
Dairy	371.06 ± 206.71	439.11 ± 251.70	492.28 ± 256.89	<0.001	0.041	<0.001	0.032
Nutrients							
Zinc (mg)	9.18 ± 3.44	8.96 ± 3.57	8.46 ± 3.96	0.333	0.411	0.505	0.620
Iron (mg)	16.32 ± 5.45	15.95 ± 5.06	15.68 ± 6.66	0.701	0.312	0.453	0.715
Calcium (mg)	956.83 ± 374.73	1046.70 ± 394.27	1009.32 ± 455.05	0.292	0.250	0.435	0.170
Vitamin C (mg)	130.02 ± 67.57	135.23 ± 74.82	139.59 ± 83.79	0.912	0.905	0.865	0.890
Vitamin D (IU)	1.67 ± 1.49	1.55 ± 1.32	1.58 ± 1.55	0.901	0.782	0.693	0.805
Vitamin B12 (mcg)	4.20 ± 2.24	4.03 ± 2.43	3.74 ± 2.14	0.373	0.320	0.471	0.690
Vitamin B2 (mg)	1.99 ± 0.76	2.04 ± 0.76	1.96 ± 0.82	0.791	0.601	0.815	0.902
Vitamin B6 (mg)	1.72 ± 0.70	1.65 ± 0.64	1.62 ± 0.71	0.501	0.705	0.581	0.675
Vitamin A (RAE)	1201.89 ± 683.68	1338.69 ± 90530	1403.65 ± 114.98	0.610	0.374	0.713	0.651

Abbreviation: DASH, dietary approach to stop hypertension. ^a^ One-way ANOVA for energy intake and ANCOVA test for other variables; (adjusted for energy intake ) and <0.05 is significant; ^b^ Tukey test; ^c^ Mean± SD. <0.01 is significant.

**Table 3:** Comparison of dietary quality indices across the DASH tertiles

**Variables**	**DASH diet**			***P*** **value fortrend** ^a^	***P*** **value for comparison T1&T2** ^b^	***P*** **value for comparison T1&T3** ^b^	***P*** **value for comparisonT2&T3** ^b^
	**T1 (n = 108)**	**T2 (n= 101)**	**T3 (n =95)**				
AHEI-2010	24.61 ± 8.01	23.04 ± 7.87	21.23 ± 8.26	0.010	0.541	0.002	0.615
DDS	5.10 ± 1.15	5.52 ± 1.25	5.47 ± 1.01	0.010	0.010	0.010	0.453
Diversity score of dairy	1.12 ± 0.61	1.36 ± 0.60	1.19 ± 0.61	0.010	0.006	0.075	0.003
Diversity score of fruits	1.54 ± 0.53	1.65 ± 0.52	1.73 ± 0.48	0.010	0.123	0.005	0.914
Diversity score of vegetables	0.69 ± 0.28	0.78 ± 0.31	0.82 ± 0.28	0.002	0.033	0.001	0.342
Diversity score of grain	1.06 ± 0.24	1.14 ± 0.26	1.10 ± 0.27	0.122	0.312	0.374	0.221
Diversity score of meat	0.67 ± 0.40	0.57 ± 0.42	0.61 ± 0.46	0.281	0.212	0.341	0.415
DDS/DED	4.4 ± 1.9	5.7 ± 2.5	6.7 ± 2.9	<0.001	0.050	<0.001	0.070
DED	1.26 ± 0.30	1.15 ± 0.27	0.99 ± 0.35	<0.001	0.020	<0.001	<0.001
MAR	1.18 ± 0.41	1.19 ± 0.46	1.17 ± 0.48	0.933	0.922	0.931	0.900

DASH, dietary approach to stop hypertension; AHEI, Alternative Healthy Eating Index; DDS, dietary diversity score; DED, dietary energy density; MAR, mean adequacy ratio. ^a^ One-way ANOVA for DED and ANCOVA test for other variables (adjusted for energy intake) and <0.05 is significant ^b^ Tukey test; ^c^ Mean± SD.

**Table 4:** Comparison of dietary intake and food groups across the tertiles of the DASH diet

**Variables**	**DASH diet**			***P*** **value fortrend** ^a^	***P*** **value for comparison T1&T2** ^b^	***P*** **value for comparison T1&T3** ^b^	***P*** **value for comparisonT2&T3** ^b^
	**T1 (n = 108)**	**T2 (n= 101)**	**T3 (n =95)**				
NARs of different nutrients							
Zinc (mg)	1.14 ± 0.43^c^	1.12 ± 0.44	1.05 ± 0.49	0.333	0.371	0.141	0.307
Iron (mg)	0.90 ± 0.30	0.88 ± 0.28	0.87 ± 0.37	0.705	0.710	0.514	0.771
Calcium (mg)	0.95 ± 0.37	1.04 ± 0.39	1.00 ± 0.45	0.294	0.305	0.440	0.613
Vitamin C (mg)	1.73 ± 0.90	1.80 ± 0.99	1.86 ± 1.11	0.641	0.550	0.601	0.634
Vitamin D (IU)	0.0028 ± 0.0025	0.0026 ± 0.0022	0.0026 ± 0.0025	0.915	0.905	0.911	0.952
Vitamin B3 (mg)	1.65 ± 0.53	1.55 ± 0.54	1.48 ± 0.64	0.090	0.084	0.112	0.096
Vitamin B12 (mcg)	1.75 ± 0.93	1.68 ± 1.01	1.56 ± 0.89	0.375	0.441	0.515	0.564
Vitamin B2 (mg)	1.81 ± 0.69	1.85 ± 0.69	1.78 ± 0.75	0.791	0.811	0.783	0.841
Vitamin B6 (mg)	0.13 ± 0.05	0.12 ± 0.049	0.12 ± 0.05	0.501	0.615	0.664	0.710
Vitamin A (RAE)	1.71 ± 0.97	1.91 ± 1.29	2.00 ± 1.63	0.605	0.554	0.540	0.701

DASH, dietary approach to stop hypertension; NAR, nutrient adequacy ratio. ^a^ ANCOVA test for variables (adjusted for energy intake) and <0.05 is significant. ^b^ Tukey test; ^c^ Mean± SD.

**Table 5:** Correlation between DASH score and dietary quality

**Variables**	**r**	***P*** **value for trend** ^*^
DDS	0.24	<0.001
AHE-I	-0.20	<0.001
DED	-0.39	<0.001
MAR	0.19	0.001
Mediterranean	0.20	0.001
NAR		
Iron	0.12	0.033
Calcium	0.23	<0.033
Zinc	0.03	0.545
Vitamin D	0.04	0.944
Vitamin C	0.17	0.003
Vitamin A	0.19	0.001
Vitamin B6	0.04	0.471
Vitamin B12	-0.03	0.602
Vitamin B2	0.14	0.010
Vitamin B3	-0.07	0.191

DASH: Dietary approach to stop hypertension, DDS: dietary diversity score, AHEI: Alternative Healthy Eating Index, DED: dietary energy density, MAR: mean adequacy ratio, NAR: nutrient adequacy ratio. ^a^The partial correlation was run to determine the interaction between DASH score and dietary quality while controlling for energy intake, age, educational level and income (<0.05 is significant).
